# Significance of High-Containment Biological Laboratories Performing Work During the COVID-19 Pandemic: Biosafety Level-3 and -4 Labs

**DOI:** 10.3389/fbioe.2021.720315

**Published:** 2021-08-13

**Authors:** Kenneth B. Yeh, Kairat Tabynov, Falgunee K. Parekh, Illich Mombo, Kyle Parker, Kaissar Tabynov, Shelton S. Bradrick, Ashley S. Tseng, Ji-Rong Yang, Lolly Gardiner, Gene Olinger, Bradly Setser

**Affiliations:** ^1^ MRIGlobal, Kansas City, MO, United States; ^2^ International Center for Vaccinology, Kazakh National Agrarian Research University, Almaty, Kazakhstan; ^3^ EpiPointe, Cary, NC, United States; ^4^ International Center for Medical Research of Franceville (CIRMF), Franceville, Gabon; ^5^ Department of Epidemiology, University of Washington School of Public Health, Seattle, WA, United States; ^6^ Taiwan Centers for Disease Control, Taipei, Taiwan; ^7^ JRAD, Stafford, VA, United States

**Keywords:** biosecurity, BSL-3, BSL-4, COVID-19, high containment biological laboratory

## Abstract

High containment biological laboratories (HCBL) are required for work on Risk Group 3 and 4 agents across the spectrum of basic, applied, and translational research. These laboratories include biosafety level (BSL)-3, BSL-4, animal BSL (ABSL)-3, BSL-3-Ag (agriculture livestock), and ABSL-4 laboratories. While SARS-CoV-2 is classified as a Risk Group 3 biological agent, routine diagnostic can be handled at BSL-2. Scenarios involving virus culture, potential exposure to aerosols, divergent high transmissible variants, and zoonosis from laboratory animals require higher BSL-3 measures. Establishing HCBLs especially those at BSL-4 is costly and needs continual investments of resources and funding to sustain labor, equipment, infrastructure, certifications, and operational needs. There are now over 50 BSL-4 laboratories and numerous BSL-3 laboratories worldwide. Besides technical and funding challenges, there are biosecurity and dual-use risks, and local community issues to contend with in order to sustain operations. Here, we describe case histories for distinct HCBLs: representative national centers for diagnostic and reference, nonprofit organizations. Case histories describe capabilities and assess activities during COVID-19 and include capacities, gaps, successes, and summary of lessons learned for future practice.

“Chance favors the prepared mind, and opportunity favors the bold.”― Louis Pasteur

## Background

The field of global health security, while diverse in its methodology and applications, can be simply delineated by the prevent-detect-respond model. Major activities are performing research and development (R&D), especially those involving international cooperation, and conducting infectious disease surveillance; both enhance global health security and specifically biosecurity by upholding the Biological Weapons Convention (BWC) and the United Nations Security Council Resolution (UNSCR) 1,540 that prohibit the use of biological weapons and are legally binding for all countries. R&D and infectious disease surveillance activities also support globally recognized frameworks such as the International Health Regulations (IHR) 2005 and the Global Health Security Agenda (GHSA), which both strengthen human and veterinary health systems. As a whole, a web of prevention model illustrates a framework of these major instruments categorized under biosafety and biosecurity (Novossiolova et al., 2019). Recent topics for dual-use research of concern and gain of function were not yet included in the framework in part due to lack of a common and consistent policy. In addition, there are guidelines for biological containment according to tiered levels for laboratories.

At its core, biological containment refers to primary containment such as equipment such as biosafety cabinets (BSC) used in HCBLs to prevent contamination of the sample of interest and protect the people handling those materials and secondary containment related to infrastructure (2, 3. High containment biological laboratories (HCBL) refer to the biosafety level-3 (BSL-3) and -4 (BSL-4) laboratories which are designed to contain pathogens to prevent their release into the environment and provide a safe setting to protect those working with these pathogens (Peters, 2018). Although the World Health Organization (WHO) and the United States (US) Centers for Disease Control and Prevention (CDC) biosafety guidelines are widely accepted, there is no standard oversight and efforts have focused on harmonizing national safety guidelines for working with pathogens (Stavskiy et al., 2003). The WHO and NIH also provide guidance for dual-use research of concern and gain-of-function studies which are often performed in HCBLs that require assurances to prevent laboratory accidents, potential misuse, and unintended consequences (National Academies of Sciences Engineering and Medicine, 2017). For this work, we refer to HCBL as those BSL-3 and BSL-4 laboratories.

Risk-based assessments continue to be objective approaches to ensure biosafety and biosecurity in HCBLs when performing tasks with agents and material such as new and emerging viruses that are not fully characterized (Meechan and Potts, 2020; World Health Organization, 2020). For example, if an agent is being studied in a region where it becomes endemic, the risk group that agent is assigned generally moves from a higher risk group level to a lower risk group level. Since countries and regions set their own risk group levels, an agent maybe assessed to have different risk in different areas as shown in one biosafety professional society’s online database (ABSA, 2021). Researchers have also suggested an interim classification for SARS-CoV-2 as a Risk Group-3 agent with BSL-3 guidelines and regulations (Kaufer et al., 2020). The International Organization for Standardization (ISO) 35001:2019 biorisk management for laboratories and other related organizations, which provides an organized framework that organizations can use to identify and manage risks especially for emerging pathogens, was released in 2019 (Callihan et al., 2021).

HCBLs play a critical role in rapid advancement of research to characterize human and animal pathogens, assist in disease surveillance, and conduct the initial pre-clinical research that sustains the pipeline for development of diagnostics, therapeutics and vaccines. Moreover, the research conducted in HCBLs enables improved understanding of the pathogen and in turn informs the necessary public health measures to control and prevent further transmission and disease pathogenesis. The scientific capabilities of the highly trained personnel working in HCBLs are a tremendous resource not only because of the research they conduct, but also because they can provide critical guidance on how to effectively manage a highly infectious pathogen at lower containment levels if necessary. The COVID-19 pandemic has demonstrated the importance of HCBLs, their associated resources, and trained personnel in combatting epidemics caused by emerging pathogens.

HCBLs have evolved in terms of infrastructure, physical controls, policies, human resources, and workforce. Biodefense in the context of countering biowarfare and bioterrorism developed and grew as a market and enterprise following September 11, 2001 and the anthrax letter attacks. In addition, interest in HCBLs has resulted in growth in number of BSL-3 and BSL-4 laboratories worldwide and subsequent oversight (Gottron, 2009). Designing, constructing and commissioning of high containment laboratories is costly and requires a large investment for operations, maintenance, sustainment, and workforce across R&D. These specialized laboratories are required for conducting basic, applied, and translational phases of research involving high-consequence pathogens (Michelotti et al., 2018). Despite operational and sustainment costs, this investment in HCBLs have helped many countries characterize emerging pathogens and control epidemics (Hottes et al., 2012).

Several motivations for building HCBLs exist including diagnostic needs, enhancing infectious disease surveillance capability and sustainability, promoting growth for life science and biotechnology, and improvements in scientific research capabilities (Hottes et al., 2012). HCBLs, unlike the lower containment levels, BSL-1 and -2, which are considered “basic” like those often found in academic institutions and hospitals, are built to be suitable for work with indigenous or exotic agents that may cause serious or potentially lethal disease (Meechan and Potts, 2020; World Health Organization, 2020). In addition, the highest containment BSL-4 laboratories are designed for work with lethal and exotic agents that pose a high individual risk of aerosol-transmitted laboratory infections and life-threatening diseases that are frequently fatal (Meechan and Potts, 2020). The goals of the CDC and WHO guidance are to ensure a safe working environment using best practices, proficient use of building and facility controls, and to provide a culture of responsibility.

Although CDC and WHO recommend that clinical samples such as confirmed and suspected COVID-19 specimens can be handled in BSL-2 facilities for routine diagnostic purpose, isolation or propagation of high concentrations of viruses should be conducted in BSL-3 at minimum (Centers for Disease Control and Prevention, 2021; World Health Organization website, 2021). The COVID-19 pandemic highlights the importance of high containment facilities, their associated resources and trained personnel needed to respond to emerging diseases. Here, we describe case histories for distinct HCBLs including national and state centers for diagnostic and reference testing located in Kazakhstan, Gabon, Taiwan, and a nonprofit organization located in the United States.

## Case Histories

While the HCBLs described may have different missions, all of them adhere to international norms and instruments discussed earlier as well as national and local regulations. Two HBCLs have bilateral partnerships with the US Defense Threat Reduction Agency’s Biological Threat Reduction Program (BTRP) which include agreements that adhere to the BWC and UNSCR 1540. The partnerships focus on biosafety and biosecurity, infectious disease surveillance and cooperative research. Our case histories describe capabilities and assess activities during COVID-19 and include capacities, gaps, successes, and summarize lessons learned. For example, one state public health laboratory in the US successfully increased laboratory capacity by cross-tasking personnel already trained in molecular extraction and who were already respirator fit-tested to work in BSL-3 (Yeh, 2020). Their capacity increased in COVID-19 testing from 100 tests/day in March 2020 to 1,700 tests/day in December 2020. Overall, the work responding to the demands of COVID-19 reflects the respective laboratory’s mission, readiness, preparation, and practice invested earlier for these assets (Table 1).

**TABLE 1:** Summary of high-containment biological laboratories (HCBL) described in case histories. HCBLs from three continents are listed.

HCBL		Organization type	Description
BSL-3	Kazakhstan Central Reference Laboratory	National laboratory	Crucial functions (Masgut Aikimbayev’s National Scientific Center, 2021)
			• Repository for especially dangerous pathogen
			• Reference, research, and training center
			• Promotes international laboratory practices
	MRIGlobal	Non-profit research	Accreditations and registrations (MRIGlobal, 2021)
			• CDC registered for Select Agent and Toxins
			• CAP, CLIA certified laboratories
			• ISO-9001:2015, ISO 17025:2005
	Taiwan Centers for Disease Control (CDC)	National Laboratory	Relevant mission (Taiwan CDC, 2021)
			• National reference and research center
			• Promotes international laboratory practices
BSL-4	International Center for Medical Research of Franceville (CIRMF)	National laboratory	Crucial roles
			• National diagnostic center and reference laboratory
			• Regional center for diagnosis of pathogens including bacteria (anthrax) and viruses: CCHF, rabies, SARS-CoV-2, Ebola, Marburg, polio (Leendertz et al., 2006; Towner et al., 2007; Grard et al., 2010; Grard et al., 2010; Grard et al., 2011; Grard et al., 2012)
			• Recognized international center for research

### National Reference BSL-3 Laboratory: Central Reference Laboratory, Almaty, Kazakhstan

As part of the legacy United States (US) Cooperative Threat Reduction program and long-standing partnership with Kazakhstan, DTRA BTRP funded and commissioned the Central Reference Laboratory (CRL) located in Almaty on the National Scientific Center for Especially Dangerous Infections named after M. Aikimbayev (NSCEDI). The CRL validation was completed in August 2017, and subsequently on September 29, 2017, the facility was transferred to the Government of Kazakhstan. Several cooperative research projects were also funded to support studies that the CRL would eventually house and perform (Yeh et al., 2019). The CRL has a modern design with both BSL-2 and BSL-3 laboratories and an ABSL-3 vivarium which reflects international norms for biosafety and biosecurity.

With its capacity, capability, and national stature, the CRL serves as the leading diagnostic laboratory in Kazakhstan. It maintains the national collection of strains of especially dangerous pathogens or EDP from the Ministry of Health (MoH) and Ministry of Agriculture (MoA) and has organizational and methodological functions including informatics and analysis typical of similar public health laboratories. The CRL facility operates under NSCEDI (MoH) management, with MoA and Ministry of Education and Science (MoES) assigned rent-free space in the facility, with dedicated BSL-2 laboratories. The MoA also shares BSL-3 and ABSL-3 laboratory space with the MoH. By agreement, MoH, MoA, and MoES are collaborating through consolidated efforts and resources. So far, over 100 CRL specialists have been trained, half of which are NSCEDI employees, 25 are MoES employees, and 25 are with MoA staff. The CRL is becoming a regional center of international cooperation for joint research, and professional training in with partners including Armenia, Azerbaijan, Georgia, Kyrgyzstan, Mongolia, Russia, Tajikistan, Turkmenistan, and Uzbekistan.

The CRL also implemented a quality management system in accordance with the requirements of ISO 9001:2015 Quality Management Systems. DEKRA (DEKRA, 2021), a German company and member of the international accreditation associations IAF and DAkkS, performed the accreditation. On July 30, 2020, the CRL was accredited according to ISO 35001:2019 Biorisk management for laboratories and other related organizations. The CRL is the first laboratory in Central Asia and one of the first in the world to be accredited under the new international standard for laboratory biological risk management, which defines requirements and recommendations for laboratories working with hazardous biological agents and materials in order to control and reduce any risks associated with their storage and use.

During the COVID-19 pandemic, DTRA funded laboratories including the CRL became active for diagnostic testing and related R&D. In May 2020, the International Center for Vaccinology at the Kazakh National Agrarian Research University (KazNARU) and the NSCEDI developed a national vaccine against SARS-CoV-2 virus infection (World Health Organization website, 2021). This is a subunit vaccine based on ESSAI O/W 1849101 from Seppic, France which is a nano-emulsion oil adjuvant for intramuscular immunization. A recombinant SARS-CoV-2 Spike protein obtained by baculovirus expression was used as an antigen. Tests of the obtained vaccine adjuvant subunit with different antigenic loads in SPF (specific pathogens free) BALB/c mice for safety and immunogenicity started on August 17, 2020. KazNARU and NSCEDI performed these studies in the CRL’s ABSL-3 laboratory. In addition to mouse experiments, the safety, immunogenicity, and protectiveness of this vaccine has been evaluated in Syrian hamsters. The National Center of Medicines and Medical Devices Expertise is planning preclinical trials for the NARUVAX-C19 vaccine in mid-2021. Concurrent vaccine safety and immunogenicity trials are planned to be conducted on non-human primates in animal models using rhesus macaques at the Research Institute of Medical Primatology in Sochi, Russia.

On August 28, 2020, KazNARU and The Ohio State University, USA also generated a vaccine based on chitosan with mannose nanoparticles for intranasal application (NARUVAX-C19 Nano). Recombinant SARS-CoV-2 Spike/RBD protein was used as an antigen. Testing of this vaccine in laboratory animals is started in mid-September 2020 in the CRL’s ABSL-3 laboratory. In early 2021, research for a hamster model to evaluate the effectiveness of SARS-CoV-2 vaccine candidates was completed. The SARS-CoV-2 hamster challenge model confirmed immunogenicity of our vaccines and verified their protective efficacy against intranasal infection with D614G spike variant of SARS-CoV-2.

On August 14, 2020, KazNARU, NSCEDI, and TreeGene LLP, used a GISAID platform (GISAID, 2021) and published the genetic sequence of the spike protein from three different SARS-CoV-2 viruses isolated in Kazakhstan. One was original material from an infected patient (hCoV-19/Kazakhstan/KazNAU-NSCEDI-481/2020, Accession ID: EPI_ISL_514093) and two SARS-CoV-2 viruses (hCoV-19/Kazakhstan/KazNAU-NSCEDI-5/2020, Accession ID: EPI_ISL_514126; hCoV-19/Kazakhstan/KazNAU-NSCEDI-7/2020, Accession ID: EPI_ISL_514127) previously isolated at NSCEDI (BSL-3 laboratory at CRL) were used as research samples. The purpose of this work was to establish which viral sequences correlate with its infectious potential. It was shown that an amino acid mutation D614G was important.

In November 2020, the isolated and characterized 3 strains (hCoV-19/Kazakhstan/KazNAU-NSCEDI-5/2020, Accession ID: EPI_ISL_514126; hCoV-19/Kazakhstan/KazNAU-NSCEDI-7/2020, Accession ID: EPI_ISL_514127; hCoV-19/Kazakhstan/KazNAU-NSCEDI-481/2020, Accession ID: EPI_ISL_514093) of SARS-CoV-2 were deposited in the Republican Collection of Microorganisms and Depository of Especially Dangerous Infections of the NSCEDI. These strains are used to test diagnostic test systems and vaccines and to determine the antiviral activity of various substances *in vitro* and *in vivo* at the CRL.

On March 19, 2021 the Minister of Health of Kazakhstan reported COVID-19 positive samples from Almaty that were found to have mutations of representing the Alpha and Beta variants of SARS-CoV-2. The International Center for Vaccinology of KazNARU and the Scientific and Practical Center for Sanitary and Epidemiological Expertise and Monitoring of the Ministry of Health of the Republic of Kazakhstan confirmed the presence of mutations in these samples. KazNARU, Kazakhstan-Japan Innovation Center, and TreeGene performed sequencing of the complete S gene. The results showed that in one sample, the S gene contained amino acid substitutions 501Y, 570D, 614G, 681H, 716I, 982A, 1118H, and amino acid deletions H69, V70, Y144, which are all typical of the Alpha variant B.1.1.7 (posted at GISAID on March 25, 2021). In the second sample, the S gene contains amino acid substitutions 80A, 215G, 242H, 417N, 484K, 501Y, 614G, 701V, 1078S, amino acid deletions A243, L244, H245, which are all typical of South African variant B.1.351 (posted on GISAID March 25, 2021). The NSCEDI and KazNARU research team isolated these virus variants in their BSL-3. Availability of these variants will allow Kazakhstani scientists to evaluate the effectiveness of both domestic and foreign vaccines and drugs against COVID-19.

### Nonprofit Organization BSL-3 Laboratory: MRIGlobal, Kansas City, Missouri, USA

For-profit companies, non-profit organizations, as well as national laboratories often employ personnel with expertise in diverse fields such as chemistry, engineering and biology who perform contract research and development, test and evaluation and related work. These organizations also invest heavily in their facilities and equipment as part of growing their core competencies to support their respective client’s project work. MRIGlobal is a non-profit research organization that provides scientific solutions in a range of disciplines to commercial and government clients. Investment in staffing resources and quality management systems provided some of the foundational core competencies that lead to MRIGlobal’s work with pathogens, obtaining a Biological Select Agent and Toxins (BSAT) registration through the US CDC’s Federal Select Agent Program, and related biosafety and biosecurity standards. All BSAT work performed at MRIGlobal is done in compliance with select agent code of federal regulations (CFR) which are 7 CFR Part 331, 9 CFR Part 121 and 42 CFR Part 73.

MRIGlobal’s BSAT program maintains compliance with the regulations described in their corporate Biological Safety Plan, Biosecurity Plan, and Incidence Response Plan (IRP). MRIGlobal also maintains numerous operational SOPs related to the handling, storage and disposal of BSATs which are regularly reviewed and updated according to guidance on BSAT. There is also a personnel reliability program employed for all staff that have access to and work with BSAT.

Prior to initiating any laboratory work with infectious biological materials, MRIGlobal requires a review and approval of the planned project work by the Biosafety Officer (BSO) and the Biosafety Review Committee (BRC). The BRC reviews all new projects involving biological materials, toxins, recombinant deoxyribonucleic acid (rDNA), or synthetic nucleic acid molecules, to determine any potential safety issues. The review includes an assessment of the agents that will be worked with, the laboratory space that will be used for the work, the relevant personnel, and their laboratory experience. In addition, a Hazard Analysis is performed to identify any potential safety issues and ways to mitigate the risk to ensure the safety of laboratory personnel.

Initially during the COVID-19 pandemic, one of MRIGlobal’s BSL-3 facilities was designated as the laboratory space dedicated to working with the SARS-CoV-2 virus. As the demand for work increased, so did the utilization of the BSL-3, which decreased the availability of Class II BSCs. For example, existing non-COVID-19 project work with BSAT and Risk Group 3 agents was being performed in parallel. According to a risk assessment for handling high and low concentrations of virus, MRIGlobal converted a BSL-2 microbiology laboratory space into a BSL-2 enhanced, or BSL-2 plus (BSL-2+), laboratory space for activities using low concentrations of virus. This is common practice in which the BSL-2+ laboratory is essentially a BSL-2 laboratory with some BSL-3 features and additional safety equipment, in this case a requirement for respiratory protection and an area for donning and doffing of personal protective equipment (PPE) were established (Centers for Disease Control and Prevention, 2021). It is also critical that the laboratory has negative pressure airflow, to ensure that aerosols do not escape the laboratory. The PPE requirements for this laboratory were updated to include the following items: safety glasses, N95 respiratory protection, back-tying gowns, shoe covers and double-layered gloves, with the inner layer of gloves taped to the sleeves of the gown. The procedure also requires staff to don respiratory protection prior to entering the laboratory. Once in the laboratory, staff don the remaining items in a “booty-free zone,” which is an area of the laboratory near the door that is marked off on the floor for entry and exit procedures. When staff exit the laboratory, they first remove shoe covers prior to entering the “booty-free zone.” Once in this area, they remove all remaining PPE, except for their respirator. They then proceed to two-step handwashing. Staff first wash their hands and exit the laboratory, where they promptly remove the respirator with a clean pair of gloves. Staff then wash their hands again prior to leaving the laboratory hallway.

Any work with SARS-CoV-2 virus or nucleic acids is performed in a BSC in the BSL-2+ laboratory to prevent exposure to the virus through spills or aerosolization. Waste materials from the BSL-2+ are designated as medical waste and are removed from the laboratory space for incineration off-site. Typically, SARS-CoV-2 materials are bleach inactivated prior to removal from the laboratory space. The implementation of these enhanced procedures and staff training resulted in the successful conversion of a BSL-2 laboratory into BSL-2+ laboratory space. This resulted in doubling the number of BSCs available for handling and processing SARS-CoV-2. MRIGlobal was able to designate this converted space for project work with the virus at near-limit of detection (LOD) levels, while work with high-titer virus stocks was reserved for the BSL-3 facility. A proper risk assessment demonstrated the BSL-2+ can serve as an effective model for laboratories looking to increase available laboratory space for use with non-BSAT infectious pathogens. Current guidance from the CDC limits these efforts at BSL2+ with newly emerging SARS-CoV-2 variants and requires work with variants to be performed at BSL-3 (Centers for Disease Control and Prevention, 2021). While this measure protects the community, it challenges the pace for more timely research and development of countermeasures.

### National Reference BSL-4 Laboratory: CIRMF, Franceville, Gabon

The primary mission of the CIRMF BSL-4 laboratory is to diagnose suspected cases of hemorrhagic fever and investigate related cases in the Central Africa region, which has experienced outbreaks of Ebola and other hemorrhagic fever pathogens. Ebolavirus disease is a hemorrhagic disease caused by one of the most feared and virulent pathogens currently threatening human population (Goldstein et al., 2018). Since its first report in the town of Yambuku in the Democratic Republic of Congo in 1976, several epidemics occurred in the human population with mortality rates reaching 90%. Since then, most of the approximately 17 outbreaks have occurred in Central Africa and viral hemorrhagic fevers gained attention widespread in the 1990s (Leroy and Gonzalez, 2012). Because of the lack of specific expertise and adequate structures in the field of viral hemorrhagic fevers, the International Center for Medical Research of Franceville (CIRMF, actually Interdisciplinary Centre for Medical Research of Franceville) became involved in the fight against Ebola infection after the second epidemic in Mayibout in 1996 (Georges et al., 1999). Hence, it was important to develop a HCBL for the development of effective and rapid specific diagnosis of viral hemorrhagic fevers and to have a backup for field investigation in endemic areas. First, the Foreign Ministry of France funded an enhanced BSL-3 plus (BSL-3+) which was built in 1997 and included negative pressure and a glove box (Leroy and Gonzalez, 2012). Similar to a BSL-2+, a BSL-3+ is a BSL-3 with features of a BSL-4. Second, a BSL-4-like laboratory for handling Risk Group 3 and 4 pathogens, was built from 2003 to 2008 at CIRMF which is one of the two BSL-4 labs throughout African continent. The Total Gabon Oil Company and Gabonese Government funded this project.

Following the Mayibout outbreak, the Emerging Viral Disease Unit (UMVE) was set up in 1998 to study the emerging Ebolavirus disease. The CIRMF BSL-4-like, which UMVE managed and operated, is now managed by the Department of Virology. Many researchers, technicians and engineers as well as agents of the Ministry of Health of Gabon have been trained and empowered to work in this laboratory. Today four people, all working at CIRMF, are the only ones authorized to enter and to work there. They are also in charge of training collaborators whose research requires protocols to be developed in high containment environments. Since the beginning of its construction, almost all suspected or sporadic cases of the human and animal epidemics from 1997 to 2019 in the Central African sub-region have been diagnosed in this laboratory. The glove box and equipment such as climatic chambers, heat chambers and incubators allowed for the isolation and the culture of many variants. Thus CIRMF maintains a stock of samples from past epidemics of Ebola virus and Marburg virus strains isolated from both humans and great apes, and strains of rhabdoviruses, Crimean Congo Hemorrhagic Fever virus and poliovirus. Nevertheless, all poliovirus strains were destroyed according to the WHO procedures in the Polio eradication program.

Because of its ability to respond against hemorrhagic fever epidemics especially Ebolavirus disease, that occurred in various countries of the Central African Region, the CIRMF BSL-4 was designated as a WHO Collaborative Centre for the diagnosis of hemorrhagic fevers in 2016–2020. Thus, the laboratory fully adhered to WHO guidelines. Today, CIRMF continues a partnership with the DTRA BTRP where Sandia National Laboratory performed a risk assessment in order to update all the guidelines regarding the biosafety and the biosafety. Further assessments are planned at CIRMF to attain ISO:35001.

On January 31, 2020, the WHO declared COVID-19 as a global health emergency after SARS-CoV-2 spread in almost all continents. Fearing a rapid spreading throughout the African continent, the Africa Centres for Disease Control and Prevention (Africa CDC) chose the CIRMF among the reference laboratories for the diagnosis of suspected COVID-19 cases. CIRMF had the infrastructure including BSL-3 and BSL-4 laboratories with the scientific expertise, reputation, and experience managing Ebola diseases and arboviruses outbreaks (Leroy et al., 2004; Mélanie et al., 2012). Thus, Gabonese Health Ministry decided to send the suspected cases to CIRMF for diagnosis. The first COVID-19 cases detected in Gabon came soon after the first case of a Gabonese traveler returning from France was reported on March 2020 (PMID: 33958601-country report). Because of the highest biosafety measures and limited access to the BSL-4-like, handling SARS-CoV-2 suspected cases for diagnosis was performed in the BSL-3 laboratory. Similar to what the rest of the world experienced during the COVID-19 pandemic, the national steering group against COVID-19 in association with the Ministry of Health in Gabon and the Gabonese government agreed to increase the daily diagnostic capacity between 10,000 and 20,000 tests for the entire country. This also increased the number of centers capable of performing the diagnosis most of them in Libreville the capital of Gabon. In this context, the BSL-4-like was included to serve as a backup laboratory in order to increase the diagnostic capacity at CIRMF if necessary. By contrast, the epidemic in Gabon was less severe. From March 2020 to May 2021 approximately 23,565 cases (most in Libreville with 14,449) have been recorded for only 143 deaths (Africa Centres for Disease Control and Prevention, 2021). Since the number of cases was less than expected, diagnosis has been well managed in the BSL-3 laboratory, the BSL-4 laboratory has not been used. Consequently, the BSL-4 laboratory did not play a role even though the people qualified to work in this laboratory trained health care workers to use category 3 Personal Protective Equipment. The laboratory remains a facility for the isolation of SARS-CoV-2 and continues to be operational for the diagnosis of any suspected cases of hemorrhagic fever.

### National Reference BSL-3 Laboratory: Taiwan CDC

As part of their mission, the Taiwan Centers for Disease Control (Taiwan CDC) has engaged with international partners to perform activities aligned with WHO and IHR for performing Joint External Evaluations of their health systems and also developed national legislature for regulating high containment laboratories, biosafety, and managing select agents and toxins (Lo, 2017; Hsieh et al., 2020; Taiwan Centers for Disease Control, 2021). In recent years, the Taiwan CDC systematically constructed a national tiered infectious disease diagnostic network, expanded infectious disease diagnostic items and capacity, and improved preparedness for emerging infectious diseases. According to their Communicable Disease Control Act, the Ministry of Health and Welfare of Taiwan categorizes notifiable diseases according to their degrees and risks of hazards including rates of case fatality, incidence, and transmission. As such, category one communicable disease include smallpox, plague, and SARS; category two include diphtheria, typhoid fever, and dengue fever; category 3 include pertussis, tetanus, Japanese encephalitis, category four include other known communicable disease or syndromes that require monitoring; and category five include other emerging diseases that are considered to cause substantial impact on human health. Taiwan CDC commissions designated laboratories to perform high-risk diagnosis in BSL-3 laboratories are responsible for the identification of these infectious diseases.

The performance of these designated laboratories was fully demonstrated during the global outbreaks of influenza A virus subtype H7N9, MERS-CoV, and Zika viruses in 2014, 2015, and 2016, respectively. The authorized laboratories are also responsible for the diagnosis of categories 2, 3, and 4 diseases in BSL-2 or BSL-2 laboratories outfitted with negative air pressure facilities (Yang et al., 2017).

The commissioned laboratories are responsible for surveillance of important communicable diseases in the community. Clinical isolates of pathogens such as influenza, enteroviruses, and tuberculosis are routinely collected by the commissioned labs and sent to Taiwan CDC for detailed genetic, antigenic, and resistance analyses. All of the laboratories in the network are qualified through annual proficiency testing, and Taiwan CDC consistently monitors their diagnostic qualifications. Taiwan CDC and local health bureaus also provide periodic training in biosafety and technical hands-on procedures. For BSL-3 laboratory workers, the minimal requirement of biosafety and biosecurity training for each new employee of microbiological laboratories is 15 hours. After the first year, employees should also receive at least a 4-h refresher training course in each of the following years. The government of Taiwan pays the diagnostic fees in full. For example, the diagnosis of some category five diseases, such as those caused by influenza A (H7N9) and SArS-CoV-2 costs 3,000 New Taiwan dollars (NT$) per test which is approximately $100 US dollars.

From this experience, Taiwan recognizes that diagnostic testing is key to containing COVID-19. At the beginning of the COVID-19 outbreak, eight medical centers with experience in emerging viral diagnosis with BSL-3 facilities were assigned as the first group of designated laboratories on January 22, 2020 after passing proficiency tests by Taiwan CDC. These eight designated laboratories and the three Taiwan CDC laboratories located in northern, central, and southern Taiwan formed the national diagnostic network and cumulatively delivered an initial diagnostic capacity of 500 tests per day (Yang et al., 2020). Taiwan CDC distributed the laboratory standard operation protocols and test reagents used for real-time RT-PCR to each designated laboratory.

During the COVID-19 global outbreak, Taiwan has further strengthened health security by implementing a strong and forward-looking diagnostic network to prevent and control outbreaks. Expanding the capacity of the laboratory network was required to meet the various diagnostic demands, such as extending travel history to other countries and enhancing screening for those who had severe complicated pneumonia, close contact with confirmed cases, passengers of airports or cruises, passengers from the Diamond Princess cruise ship, and three Wuhan special charter flights (Taiwan MOH, 2021).

A second group of designated laboratories included three more BSL-3 laboratories and an additional 34 BSL-2 laboratories with negative air pressure facilities—were recruited for the response. A comprehensive diagnostic network with 60 laboratories was completed nationwide with a diagnostic capacity of 7,342 tests per day. In Taiwan, between January 14 and August 5 in 2020, 82,660 cases were reported, and 476 COVID-19 cases were confirmed by designated laboratories, including 7 deaths. Up to August 5, a total of 158,772 diagnostic tests were conducted (Yang et al., 2020).

COVID-19 is a category five notifiable disease in Taiwan which requires all cases fulfilling this requirement be reported to Taiwan CDC within 24 h. The turnaround time for laboratory testing results are provided in 24 h to isolate patients and trace close contacts in a cost-effective manner. COVID-19 control measures and patient management involve admitting confirmed patients in negative pressure isolation wards, home quarantine wards, and home isolation wards. These measures rely on laboratory testing—the timeliness of diagnosis results is therefore crucial for rational allocation of isolation wards and quarantine shelters and efficient use of medical resources, which can alleviate the burden on healthcare services. Proactive and targeted laboratory diagnosis of SARS-CoV-2 in Taiwan has proven to be efficient for real-time case recognition, contact tracing, and isolation. Due to the high infectivity of COVID-19 and the large proportion of patients with minor symptoms, laboratory testing with a 24-h turnaround time has been highly efficient in initiating a rapid control response. As of July 19, 2021, a network comprising a total of 226 designated laboratories conducting SARS-CoV-2 diagnosis was successfully established.

The Taiwan National Laboratory Network, which is experienced in dengue fever, avian influenza, MERS-CoV, and Zika diagnostic responses, faced a new challenge to meet the surge in demand for COVID-19 diagnostics. Laboratory surveillance needed to be further strengthened to prevent the risk of community transmission. To prepare for possible large-scale community outbreaks in the near future, further laboratory capacity will include more BSL-2 laboratories. In addition, more automatic high-throughput real-time PCR platforms as well as rapid nucleic acid and serological diagnostic tools will be integrated to expand screening capabilities. The advanced deployment of laboratory capacity for molecular diagnosis of SARS-CoV-2 made early recognition of COVID-19 cases possible and contributed to the containment and delay of community transmission in Taiwan. This strengthened laboratory capacity will help prepare for future challenges related to other emerging infectious diseases.

## Conclusion

Our case histories of four HCBLs demonstrate their application, intent, design and utility to support laboratory diagnostic and reference activities and respond to surge demands such as COVID-19. Operating costs for HCBLs are high and industry experts estimated annual budgets for maintenance costs are 10–15% of the HCBL construction cost (Heckert et al., 2011). For example, the Kazakhstan Central Reference Laboratory commissioned in 2017 cost $102M, with an annual maintenance budget estimated at $10–15M. Earlier analysis demonstrated that a balance of standardizing practices between local procedures and accepted international standards is required for integrating new HCBLs into a country’s infrastructure and into the overall scientific community worldwide (Yeh et al., 2016). Here, the laboratory investments including quality management systems such as ISO:35001 and training whether public or private funded appear justified and there is a track record for sustainable operations.

The successes of HCBLs and their trained personnel during the COVID-19 pandemic include several advancements of research and development, including supporting test and evaluation for laboratory diagnostics, vaccine studies, and related applications. The Kazakhstan Central Reference Laboratory, which is one of the newest BSL-3 laboratories, has performed impactful studies to support domestic vaccine development. Several international collaborations highlight their work that helps advance their products. MRIGlobal has supported test and evaluation for various government and commercial partners to aid in obtaining regulatory clearances for devices and assays. All of our case history laboratories also demonstrated laboratory diagnostic capabilities and ability to scale up operations during surge demands for COVID-19 testing.

In addition to increasing their capabilities and capacities, these HCBLs also adapted during the pandemic to meet changing demands during crisis moments. MRIGlobal and the Taiwan CDC cited BSL enhancements as one way to adapt existing laboratory infrastructure and increase capacity. Experts performed risk-based assessments prior to working in the BSL-2+ laboratories where they implemented modifications as well as additional training on PPE requirements and containment policies. CIRMF also had prior experience performing work in BSL-3+ and their BSL-4 was not fully used to support COVID-19.

The Taiwan CDC example highlights past preparations including avian influenza and MERS-CoV and a strong tiered network of laboratories. The capacity and capabilities within their BSL-3 laboratories provided guidance for laboratory networks at lower containment levels. The initial assessment of pathogens in BSL-3 also enabled the establishment of procedures to handle such pathogens at a lower containment level. These outputs serve as a model for pandemic response and mitigation.

While these case histories are a small representation of HCBL work, greater efforts are needed that continue to bring awareness and encourage transparency. The number of HCBLs continues to increase, that trend will likely continue with COVID-19 worldwide as countries and states will choose to prioritize and build them. Since many academic and private laboratories are not under their governmental oversight, it is difficult to obtain accurate counts of HCBLs (Peters, 2018). In our references, we also noted inconsistencies in the HCBLs especially those listed in the BSL-3 category.

While the value of HCBLs is established, the uncertain number of HCBLs also makes it difficult to ascertain capabilities and capacities for future preparedness. Regarding new and emerging pathogens, there is a need for higher biological containment for samples obtained from space exploration. These samples require containment to preserve their integrity while avoiding native Earth contamination of the sample and also to protect the Earth from extraterrestrial non-restricted or restricted bodies depending on where the sample is collected (Calaway et al., 2018). Samples from non-restricted bodies such as the Moon, asteroids, comets, solar particles and space dust require facilities that meet BSL-3 conditions. While certain samples brought to Earth from a planet that may have environments that can support microbial life are considered Category V body that must be curated at BSL-4 in accordance with the Article IX of the Outer Space Treaty. To achieve this, the samples are stored in conditions, similar to aerosol chambers, with specific gaseous atmospheres. A mobile modular BSL-4 negative pressure ISO class 5 cleanroom sample receiving facility is planned for these missions (Calaway et al., 2018). With the increasing number of HBCLs, additional challenges for sustaining operations include operation, maintenance, and further upkeep of aging facilities. Some HBCLs especially those at the top-tier of national diagnostic and reference laboratories are connected with infectious disease surveillance programs that become further important as those surveillance programs intersect with tracking and predicting patterns and trends of infectious disease to augment preparedness.

## Data Availability

The original contributions presented in the study are included in the article/supplementary material, further inquiries can be directed to the corresponding author.
